# Status and frontiers of Fabre disease

**DOI:** 10.1186/s13023-025-03646-y

**Published:** 2025-03-13

**Authors:** Wei Chu, Min Chen, Xiaoqin Lv, Sheng Lu, Changyan Wang, Limin Yin, Linyan Qian, Jiana Shi

**Affiliations:** 1 Department of Pharmacy, The First People’s Hospital of Huzhou, The Directly Affiliated Hospital of Huzhou Teachers College, Huzhou, China; 2 Center for Clinical Pharmacy, Cancer Center, Department of Pharmacy, Zhejiang Provincial People’s Hospital (Affiliated People’s Hospital, Hangzhou Medical College), Hangzhou, China; 3 Department of Pharmacy, The First People’s Hospital of Aksu District, Aksu, China; 4 Department of Drug Monitoring and Evaluation, Zhejiang Center for Drug and Cosmetic Evaluation, Hangzhou, China; 5 Department of Clinical Laboratory, Huzhou Aishan Hospital of Integrated Chinese and Western Medicine, Huzhou, China; 6 Department of Pharmacy, First People’s Hospital of Wenling, Wenling, China; 7 Heart Center, Department of Cardiovascular Medicine, Affiliated People’s Hospital, Zhejiang Provincial People’s Hospital, Hangzhou Medical College, Hangzhou, China

**Keywords:** Fabry disease, Therapeutic drugs, ERT

## Abstract

Fabry disease is characterized by an X sex chromosome gene mutation caused by α-galactosidase A deficiency, resulting in the accumulation of globotriaosylceramide and globotriaosylsphingosine in various organs, which induces end-organ lesions. In Fabry disease, enzymes with lost or decreased activity in the body are replaced by exogenous supplementation of normal-function α-galactosidase A. Currently, agalsidase α and agalsidase β are widely used for ERT therapy. However, this therapy has limitations such as high cost, short half-life, and production of neutralizing drug antibodies. The use of Migalastat as chaperone therapy has been approved in many countries, and it plays a therapeutic role by enhancing enzyme activity. However, companion therapy drugs are only suitable for patients with decreased enzyme activity, so the scope of their application is limited. In addition, there are several therapeutic drugs in development, including a new generation of ERT therapies, drugs resistant to neutralizing anti-drug antibody drugs, and substrate reduction therapy drugs. Due to the limitations of existing therapeutic drugs, researchers have begun to explore new therapeutic drugs for Fabry disease, so new pathogenic mechanisms and adjuvant therapeutic drugs have been continuously discovered, and the development of related drugs will contribute to disease control and treatment. This article summarizes the existing and potential drugs for treating Fabry disease to facilitate the selection of suitable and effective drugs for treatment.

## Background

Fabry disease (FD) is a rare X-linked genetic lysosomal storage disorder. Mutation of the α-galactosidase A gene (GLA) on the X sex chromosome results in a defect in the α-galactosidase A (AGAL) encoded by GLA, which cannot degrade globotriaosylceramide (Gb3) and then causes a large amount of Gb3 and its derivative globotriaosylsphingosine (lysoGb3) in the kidney, heart, nerve, skin and other large amounts of storage, eventually causing corresponding tissue and organ deposits.

The treatment for Fabry disease generally combines symptomatic treatment with etiological treatment. Fabry disease symptomatic treatments include pain relief medications such as pregabalin, cardioprotective medications like ACE inhibitors, nephroprotective strategies involving ACE inhibitors or angiotensin receptor blockers, among others. Specialized treatment involves the treatment of corresponding tissue and organ lesions caused by FD, which can only resolve short-term pathological symptoms, and there is still a high risk of recurrence before FD can be controlled. Causative treatments, such as restoring GLA, improving the function of AGAL and reducing the accumulation of Gb3 and lysoGb3, are targeted according to its pathogenesis.

## Fabry’s therapeutic drugs based on the Gb3 mechanism

### Marketed therapeutic drugs

#### Enzyme replacement therapy (ERT)

ERT degrades Gb3 by supplementing AGAL, which has a normal function; reducing the accumulation of Gb3 and lysoGb3 in vivo; and treating plants with two types of drugs currently used for ERT, namely, agalsidase α and agalsidase β. Although they are genetically identical, structurally, and functionally similar, and have the same amino acid sequence as natural human AGAL, they also exhibit differences.

Agalsidase α is an AGAL produced from a human cell line (the human fibrosarcoma cell line HT-1080), and agalsidase β is an AGAL produced from Chinese hamster ovary (CHO) cells, both of which can be used as exogenous AGAL to degrade the accumulated Gb3 in vivo, with similar efficacy. However, studies have shown that in male patients with typical FD, the effect of agalsidase β on reducing lysoGb3 is more obvious [1]. Although there was no difference in the treatment endpoint [2], agalsidase β can reduce lysoGb3 accumulation more quickly, thus improving the symptoms of the affected tissues more quickly. Compared with agalsidase α, agalsidase β has a greater risk of neutralizing anti-drug antibodies (ADAs) [3] and a greater incidence of infusion reactions. According to the instructions for the two drugs, the incidence of infusion reactions of agalsidase β was 67%. The incidence of agalsidase α infusion reactions was 13.7%, and the incidence of agalsidase α infusion reactions in subsequent clinical trials was 23.5%. Although CHO cells are more mature, they still have a greater risk of immunogenicity than human cells. Therefore, the risk of ADAs production and the incidence of the infusion reaction of agalsidase β derived from CHO are significantly higher than those of agalsidase α derived from human cell lines. Finally, in terms of the applicable population, according to the instructions of the two drugs, agalsidase α is suitable for adults, children, and adolescents, but its effectiveness and safety have not been established in children aged 0 to 6 years. Agalsidase β is suitable for adults, children over eight years of age and adolescents. The median ages of symptom onset were 7.0 and 9.0 years for children and 21.0 and 31.0 years for adults during the periods 2001–2006 and 2007–2013, respectively [4]. For this subset of children, agalsidase α is recommended for children under eight years old or more than eight years old with mild symptoms. Due to the lower antibody incidence and infusion reaction rate of agalsidase α, adult patients with mild symptoms may also be prioritized for agalsidase α.

Currently, ERT is the predominant approach for the management of FD, however, this method is not without its constraints. When a patient’s organs have suffered severe damage, the effectiveness of ERT may be significantly reduced. Infusion reactions, such as headache, paresthesia, hypotension, fever, chills, nausea, and fatigue, are the most common adverse effects to ERT. However, the occurrence of infusion reactions can be reduced by slowing the infusion rate and using nonsteroidal anti-inflammatory drugs, antihistamines, or glucocorticoids [5]. According to the instructions for both agalsidase α and agalsidase β, ERT requires intravenous injection every two weeks.The plasma half-life of ERT drugs is short, regular intravenous ERT can be fairly invasive to patients affecting their quality of life and impacting their psychosocial health. FD patients need lifelong treatment, and two-week treatments are too frequent for them, especially for children or teenagers; maintaining regular medication can be difficult. Furthermore, there is a high cost of the drug, which requires approximately $200,000 per year for treatment. ERT therapeutic drugs are exogenous recombinant proteins, and long-term infusion triggers the immune response, resulting in the production of specific antibodies against this enzyme [6]. ADAs not only inhibit endogenous enzymes but also the activity of exogenous input enzymes [7], resulting in the reduction or even disappearance of the therapeutic effect of ERT. Studies have shown that the likelihood of producing this antibody is also very high, with 46% of patients developing this antibody within three months of ERT treatment [8]. Even when AGAL is modified to mask immune epitopes, it still induces the production of this antibody [9]. Although the original therapeutic effect can be maintained by increasing the dose of AGAL [10], there is no clear conclusion on the optimal dose, and such a practice will waste more drug resources and cause greater economic pressure.It is important to monitor the drug interactions between ERT and other medications, and avoid using it concurrently with drugs that inhibit AGAL activity.

#### Chaperone therapy drugs

These drugs are equivalent to AGAL sensitizers, which play a therapeutic role by enhancing or restoring the activity of endogenous AGAL and the degradation of Gb3 [11]. Therefore, such drugs are only suitable for patients with decreased enzyme activity. Migalastat is a representative drug marketed in Canada, Europe, and other countries [12]. Migalastat selectively and reversibly binds to structurally and functionally defective αGal A to stabilize the protein conformation and aiding its proper folding for normal function. It increases or restores αGal A activity and facilitates its transport to lysosomes to remove stored substrates [13]. Compared with ERT, the oral administration of Migalastat can enhance the medication compliance of patients. On the other hand, it can cross the blood-brain barrier, enhance the activity of AGAL and reduce the level of Gb3 in the brain [14]. Therefore, FD patients with central nervous system symptoms may benefit from treatment with Migalastat.

Migalastat cannot produce normal functional AGAL but can only enhance enzyme activity [15]. It can only be used to treat FD patients with decreased enzyme activity. AGAL protein activity decreases, and GLA gene mutations that are restored after Migalastat treatment are called ‘amenable’ mutations. At present, there are more than 1000 known GLA gene mutations and no clear data on the proportion of ‘amenable’ mutations. However, it is estimated that ‘amenable’ mutations account for 35–50% of the total gene mutations [16, 17], which has led to the narrowing of the scope of use of Migalastat, resulting in the need to identify the GLA gene mutation type in patients before using Migalastat and increased costs and time associated with genetic testing.

### Therapeutic drugs in the research stage

#### A new generation of ERT therapeutic drugs

Based on ERT therapeutic drugs, AGAL can be modified by the covalent crosslinking form of polyethylene glycol (PEG) to obtain more stable proteins and improve its pharmacokinetic and biological distribution characteristics. This modification does not alter the tertiary protein structure of the enzyme but can enhances its stability of the enzyme while masking immune epitopes [18]. The safety and efficacy of PEG-AGAL were demonstrated in a 6-year multicenter study in adults with FD [19]. The therapeutic purpose can still be achieved even if ERT has been used and ADAs are produced in vivo [9], possibly because PEG-AGAL has a lower affinity for ADAs than AGAL [20], suggesting that PEG-AGAL treatment has a lower risk of antibody production and a stronger ability to resist antibodies.

#### Immunomodulators

Lenders et al. demonstrated the safety and effectiveness of this method by using an immunosorbent to specifically adsorb ADAs from serum while not affecting other antigens [21]. However, this method is difficult to apply in practice because antibody recovery is very fast, and more frequent immunoadsorption is needed. In addition, the effects of anti-ADAs through immunosuppression have also been studied. Although this method is effective, it does not provide long-term protective effects [22]. Some researchers have learned from the experience of Pompe disease. Banugaria et al. used a bortezomib-based immunomodulatory regimen that showed good tolerance and safety to cope with the high persistent antibody titers arising from treatment [23, 24]. Julien et al. responded to the production of antibodies in ERT using immune tolerance induction, including taking rituximab weekly for four weeks, methotrexate three times a week for three weeks, and monthly intravenous immunoglobulin injections [25]. Although the safety and efficacy of these regimens require further verification, it is still worth learning from them, combining them with ERT to reduce the possibility of ADAs antibody production and applying them to ERT treatment of FD patients who have already developed antibodies.

#### Substrate reduction therapy drugs

Substrate reduction therapy reduces the accumulation of Gb3 in various tissues and organs by inhibiting the synthesis of Gb3. Lucerastat is a low-molecular-weight iminosugar that inhibits glucosylceramide synthase, thus blocking the biosynthesis of glycosphingolipids, including those that accumulate in FD [26]. Compared to ERT, Lucerastat can be administered orally, greatly increasing patient compliance. A retrospective study showed that oral administration of the drug was not affected by food, and the occurrence of adverse reactions was independent of the dose, indicating that the drug was well tolerated [27]. Although the sample size in this retrospective study was small, it further provided a basis for using lucerastat to treat FD. According to the results of pharmacokinetic studies in rats, dogs and healthy subjects, the primary route of lucerastat clearance is the kidney, and one of the main organs involved in FD is the kidney. Although lucerastat is well tolerated, the dose of this drug needs to be adjusted for patients with moderate and severe renal impairment [28]. In the heart, one study demonstrated that large doses of lucerastat did not lead to a prolonged QT interval, nor did they have any clinically relevant effects on other electrocardiogram parameters [29]. This will reduce the risk of drug use in the overall benefit-risk assessment of FD.

#### Gene therapy

Gene therapy is based on introducing DNA carrying the genetic code for the AGAL protein into the patient’s cells, replacing the mutated GLA gene with a normally active AGAL. Gene therapy is a promising treatment that could fundamentally treat FD. At present, several gene therapies have entered clinical trials and are expected to potentially cure FD completely in the future.

Stem cell gene therapy.

Hematopoietic Stem/Progenitor Cell (HSPC)-mediated gene therapy: This treatment method often uses Lentiviruses to deliver the normal GLA gene to autologous HSPCs obtained from the patient’s body, which are then re-infused into the patient to treat FD. This approach has the effect of long-term improvement in disease progression and preliminary shows good safety. Lentivirus-mediated gene therapy is a way to deliver genes in vitro. According to the study of Aneal Khan [30], they introduced the GLA gene with an optimized human codon into autologous stem cells through recombinant lentivirus and transplanted autologous stem cells into FD patients. The recombinant lentivirus could activate the GLA gene and start the encoding of the AGAL protein by itself. This method can deliver functional enzymes to locations that ERT cannot reach and can continuously produce active AGAL protein, which effectively reduces the levels of Gb3 and lyso-Gb3 for a long time. Of the five FD patients in their study, three stopped ERT, demonstrating the safety and effectiveness of this approach.

#### Liver directed gene therapy

The original strategies for liver-directed gene therapy include replacing missing gene products, overexpressing intrinsic or extrinsic genes, and inhibiting the expression of specific genes. Viral vectors such as adeno-associated viruses (AAV) are widely studied due to their efficient targeting of the liver, lack of requirement for genomic integration, and low immunogenicity. Adenovirus-mediated gene therapy is a method for delivering genes in vivo [31]. An adenovirus containing the human codon-optimized GLA gene was injected directly into FD patients, where it targeted liver cells, produced a functional AGAL protein through the liver, and was secreted into the blood. AGAL protein levels increase in a dose-dependent manner, while Gb3 and lyso-Gb3 can decrease to normal levels. At present, this method has been used in clinical studies and has shown good gene delivery ability [32].In September 2024, the FDA granted Orphan Drug Designation to AMT-191. AMT-191 is an investigational AAV5 gene therapy that delivers a GLA transgene designed to target the liver to produce GLA protein.

#### Messenger RNA(m-RNA)-based gene therapy

m-RNA, as a novel drug modification and delivery vehicle, has been explored as a potential therapeutic means to restore or replace different types of therapeutic proteins [33]. m-RNA encoding the human GLA gene is transmitted into the body by intravenous injection, and a single injection increases AGAL protein levels and activity while decreasing Gb3 and lyso-Gb3 levels in tissue and plasma. Moreover, the half-life of the AGAL protein in tissues and plasma is extended, and the duration of substrate reduction can last up to six weeks. Continuous intravenous injection can maintain the sustained pharmacological effects of mRNA encoding the GLA gene [34, 35]. It is not a one-size-fits-all approach, but it is less immunogenic and easier to deliver than virus-based gene therapy. Moreover, this method is mature and has been widely used in the clinical production of therapeutic proteins, and COVID-19 vaccines are produced in this way [36]. Therefore, mRNA-based gene therapy is expected to become a gene replacement therapy for FD.

Compared to DNA therapy, mRNA therapy does not pose the risk of insertional mutagenesis, which is an advantage. However, this risk associated with DNA therapy remains a consideration in gene therapy.The initial costs of research and development and application may be very high, which could limit their widespread adoption and accessibility. Technological challenges include how to effectively deliver therapeutic genes to target cells and how to ensure the stability and safety of gene expression.

#### A novel method for producing α-galactosidase A—Moss-AGAL

Moss-AGAL is produced by bryophytes [37]. Unlike agalsidase α, which is highly heterogeneous, Moss-AGAL has a unique N-sugar chain and is highly homogeneous. Although sugar chains play an important role in regulating the antigenicity of therapeutic proteins [38], Moss-AGAL, with its unique N-sugar chain, may lead to different immune responses in humans than agalsidase α or β. Compared with traditional agalsidases, Moss-AGAL can be more effectively absorbed by vascular endothelial cells or other cell types [39], suggesting greater bioavailability. In addition, the targeting of Moss-agal to the kidney was significantly enhanced, while its transport to the liver was significantly reduced [39]. Although the mechanism underlying the difference in tissue distribution is unclear, it may be more appropriate to use Moss-AGAL for ERT in patients with Fabry kidney disease. Finally, Finally, Moss-agal is more cost-effective due to its lower production cost, simpler synthesis pathway, and easier operation.

## Fabry’s novel ideas for therapeutic drugs

The pathogenesis of FD involves a GLA gene mutation, which causes the activity of the encoded AGAL protein to decrease so that it cannot reduce Gb3, resulting in the accumulation of Gb3 and lyso-Gb3 in the body to induce tissue lesions. Research on drugs for treating FD is also conducted based on the pathogenesis. For example, gene therapy involves the introduction of human-optimized GLA genes to replace mutated GLA genes; ERT involves the exogenous supplementation of the AGAL protein, which has a normal function; adding Migalastat to companion therapy enhances AGAL protein activity, and substrate reduction therapy reduces the production of Gb3 from the source. Due to the limitations and shortcomings of these therapeutic methods, new FD treatment drugs are being explored. With further research, some new mechanisms for the induction of tissue deposits by FD have been discovered, offering novel insights for the study of FD treatment drugs. Drugs targeting these new mechanisms are anticipated to become new therapeutic options for the treatment of FD.

### Anti-Syn α-accumulating drugs

According to relevant studies, in patients treated with ERT, although the level of Gb3 in podocytes decreased, it did not improve podocyte injury, suggesting that nonsubstrate mechanisms continue to cause injury to end organs [40, 41]. On this basis, Fabian Braun found that Synuclein α is the key to podocyte damage through transcriptome linkage mapping and proteomics and confirmed that the accumulation of Synuclein α is the cause of FD-induced podocyte injury. Knockout and pharmacological inhibition of synuclein α can improve the lysosomal structure and function of podocytes, and this effect is even stronger than that of ERT [42]. This also explains why a damaged glomerulus cannot be cured and only slows its progression. Therefore, anti-Syn α-accumulating drugs may represent a new direction for Fabry treatment, especially for patients with Fabry kidney disease.

### Additional pathogenic pathway drugs of the Fabry non-GB3 mechanism

Elsaid cleverly utilized a zebrafish model that does not produce GB3 to investigate other potential therapeutic targets for Fabry disease, particularly the implications of the Sod2(superoxide dismutase 2) activity mechanism [43]. They observed the downregulation of proteins related to lysosomes and mitochondria and interference with energy-related pathways, as well as changes in mitochondrial morphology and function, in the GLA knockout group. The disruption of crest morphology and the reduction in the surface area of the coronoid process, which is crucial for chemical reactions in mitochondria, all contribute to reducing mitochondrial function. This also explains why FD patients are prone to fatigue and exercise intolerance. In addition, a decrease in Sod2 activity was also observed, and a similar change in Sod2 activity was observed in a study of human FD patients [44]; however, it was proposed that the decrease in Sod2 activity was not related to Gb3 accumulation, suggesting that the change in Sod2 activity is a new pathway independent of Gb3. It should be noted that the studies of these new pathways were all conducted in zebrafish. The GLA gene of zebrafish is located on the autochromosome, while the GLA gene of humans is located on the X sex chromosome. Whether this difference will lead to changes in a new mechanism requires further research.

## Fabry adjunctive therapy drugs

### Pentosan polysulfate

AGAL protein activity is decreased or even lost in FD patients, and a deficiency of this enzyme can cause lysosomal dysfunction [45]. At the same time, the production of high levels of inflammatory factors is characteristic of lysosomal diseases [46]. In addition, studies have detected high levels of inflammatory factors in FD patients [47]. Although the use of ERT and other treatments can reduce the level of Gb3, it cannot reverse the loss of end organs, suggesting that the production of inflammatory factors may be one of the causes of organ damage. Andrea took inspiration from drugs used to treat mucopolysaccharidoses [48] and discovered a compound called pentosan polysulfate (PPS). Although its anti-inflammatory mechanism is not fully understood, it has been shown to reduce NF-κB (nuclear factor kappa-light-chain-enhancer of activated B cells) activity. In an in vitro Fabry model, PPS effectively reduced the production of inflammatory factors and slowed the progression of Fabry’s kidney disease [49]. Therefore, inflammation treatment by PPS may become an adjunctive therapy for Fabry’s kidney disease.

### Fasudil

The accumulation of Gb3 in vascular endothelial cells in FD patients induces vascular lesions, including angiokeratomas [50]. These vascular lesions can further progress to other tissue lesions and are associated with related complications, such as left ventricular hypertrophy, renal failure, and stroke [51]. Therefore, Choi JB identified the role of fasudil in Fabry-induced vascular lesions by screening preclinical compounds and clinical compound libraries [52]. Fasudil can reduce p-Smad2 and thrombocytopretin-1 levels in Fabry vascular endothelial cells, increase the levels of angiogenic factors and endothelial nitric oxide synthase, inhibit the transformation of endothelial cells into mesenchymal cells induced by transforming growth factor-β, and reduce reactive oxidative stress in vascular endothelial cells. Finally, it can protect Fabry vascular endothelial cells and reduce pathological changes in the heart, kidney and other tissues, and this effect was verified in subsequent mouse experiments. Therefore, fasudil is likely to become an adjunctive therapy for cardio-cerebrovascular diseases caused by Fabry in the future.

### Glutathione

Kim JW, based on GLA gene knockout in human inducible pluripotent stem cells, used transcriptional analysis to reveal that glutathione may be a mechanism of Fabry-induced disease [53]. GSH is an endogenous low-molecular-weight mercaptan compound that plays an important role in cellular redox reactions and can protect cells from damage by alleviating oxidative stress caused by reactive oxygen species. In an in vitro Fabry model, GSH levels were reduced, and oxidative stress caused by reactive oxygen species was enhanced, leading to cell damage. By supplementing GSH, the cell damage caused by Fabry can be treated, confirming the role of GSH in Fabry-induced disease. Although current research on glutathione therapy for Fabry primarily focuses on the cellular level, it still provides a new mechanism for the cell damage caused by Fabry.This opens up new possibilities for the selection of therapeutic drugs for Fabry. GSH can be used as an adjunctive therapy to protect end organs from Fabry-induced damage.

## Conclusion

The therapeutics for Fabry disease that are currently on the market or in clinical studies have been developed based on the known pathogenesis, which includes changes at the genetic level, supplementation or activation of the α-galactosidase A (AGA) protein, and reduction of pathogenic substrates.Current treatments, ERTs have shown clinical benefits in managing symptoms and improving quality of life for affected individuals. However, these treatments have limitations, including the need for frequent infusions, high costs, and incomplete reversal of disease pathology. Future research directions for ERTs include developing more cost-effective production methods to broaden patient access, investigating strategies to minimize immune responses through enzyme modification or immune tolerance induction, and exploring formulations and delivery systems that can prolong the therapeutic effect.

Recent developments in mRNA therapy offer a promising alternative approach. Preclinical studies have demonstrated the potential of systemic mRNA therapy to produce functional enzymes, reduce substrate accumulation, and achieve sustained therapeutic effects with less frequent dosing. This approach has shown significant efficacy in animal models, with prolonged duration of action and reduced immune responses compared to traditional ERTs. Future research should focus on further optimizing mRNA delivery systems, evaluating long-term safety and efficacy in clinical trials.

Consequently,, researchers have begun to explore the novel mechanisms of tissue damage caused by FD, leading to the discovery of α-synuclein, mitochondrial mechanism, the Sod2 pathway and other mechanisms that contribute to cell damage, thereby revealing the causes of end-organ damage induced by FD.Additionally, the emergence of adjunctive therapy drugs also addresses the issue that existing drugs cannot improve damaged cells and tissues. In the future, the combination of multiple drugs is expected to improve the treatment of Fabry and ameliorate tissue damage.

## Data Availability

Not applicable.

## Abbreviations

FD: Fabry disease
GLA: α-galactosidase A gene
AGAL: α-galactosidase A
Gb3: Globotriaosylceramide
lysoGb3: Globotriaosylsphingosine
ERT: Enzyme replacement therapy
CHO: Chinese hamster ovary
ADAs: Anti-drug antibodies
PEG: Polyethylene glycol
PPS: Pentosan polysulfate
ACE: Angiotensin-Converting Enzyme
Sod2: Superoxide dismutase 2
NF-κB: Nuclear factor kappa-light-chain-enhancer of activated B cells
